# Data of XPS in incorporating the platinum complexes dopant on the surface of Ag_3_PO_4_ photocatalyst

**DOI:** 10.1016/j.dib.2019.104988

**Published:** 2019-12-12

**Authors:** Uyi Sulaeman, Richo Dwi Permadi, Dian Riana Ningsih, Hartiwi Diastuti, Anung Riapanitra, Shu Yin

**Affiliations:** a Department of Chemistry, Jenderal Soedirman University, Purwokerto, 53123, Indonesia; b Institute of Multidisciplinary Research for Advanced Materials, Tohoku University, Sendai, 980-8577, Japan

**Keywords:** Ag_3_PO_4_, Photocatalyst, Deconvolution, Defect, Platinum complexes, XPS peak

## Abstract

These data inform about the XPS profile of Ag4d, P2p, and O1s from the samples of Ag_3_PO_4_, defect-Ag_3_PO_4_, Ag_3_PO_4_/PtCl_6_^2−^ and defect-Ag_3_PO_4_/PtCl_6_^2−^ which were denoted as AP, DAP, AP/Pt, and DAP/Pt, respectively. These samples were made by co-precipitation method using the starting material of silver nitrate (AgNO_3_), disodium hydrogen phosphate dodecahydrate (Na_2_HPO_4_.12H_2_O), and hexachloroplatinic acid hexahydrate (H_2_PtCl_6_.6H_2_O) for platinum complexes dopant. The water solution and mixed water-ethanol solution for dissolving the AgNO_3_ were used for free-defect and defect samples, respectively. The Ag4d, P2p, and O1s of these samples were investigated using the XPS. The deconvolutions of O1s peak were analyzed using the software of XPSPEAK Version 4.1. The modification of Ag_3_PO_4_ by defect and platinum complexes dopant had changed the curve profile of Ag4d, P2p and O1s. Two types of oxygen of O-1 and O-2 were observed in O1s spectrum. The ratios of O-2/O-1 with the value of 0.25, 0.32, 0.49 and 0.51 were found in the sample of AP, DAP, AP/Pt, and DAP/Pt, respectively. These data are related to the research article “The surface modification of Ag_3_PO_4_ using anionic platinum complexes for enhanced visible-light photocatalytic activity” [1].

**Table:** Specifications table

Subject area	Materials Science
Specific subject area	Materials Chemistry
Type of data	Figures and Table
How data were acquired	The samples were investigated using the XPS instrument (Perkin Elmer PHI 5600). To obtain the parameter that indicated the character in percentage for each contained element, the XPS data analysis was continued by subtracting the background using Shiley method and curve-fitting the obtained signal using Gauss-Lorentz method [2]. The peak energies were calibrated by internal referencing of the adventitious carbon at 284.6 eV.
Data format	Raw and analyzed data
Experimental factors	Different conditions of the co-precipitation method. Four conditions of co-precipitation resulting in samples of Ag_3_PO_4_, defect-Ag_3_PO_4_, Ag_3_PO_4_/PtCl_6_^2−^ and defect-Ag_3_PO_4_/PtCl_6_^2−^ with the sample names of AP, DAP, AP/Pt, and DAP/Pt.
Experimental features	Identification of spectra energies profile (Ag4d, Ag3d, P2p, O1s), determination of binding energy, and deconvolution of peak energy (O1s).
Data source location	Department of Chemistry, Jenderal Soedirman University, Purwokerto, 53123, Indonesia.
Data accessibility	With the article
Related research article	*Sulaeman* et al. *“The surface modification of Ag*_*3*_*PO*_*4*_*using anionic platinum complexes for enhanced visible-light photocatalytic activity”, Mater. Lett. 259, 126848 (2020)*

**Table:** 

**Value of the Data** • The different XPS profile due to a defect and dopant incorporation on the surface of Ag_3_PO_4_ photocatalyst. • The researchers can develop Ag_3_PO_4_ properties using the defect and dopant principle. • The data can be used as a model in the improvement of photocatalytic activities by a defect and dopant treatment. • The data can be used as a model in computational chemistry in terms of defect and dopant properties.

## 1 Data

**Fig. 1:**
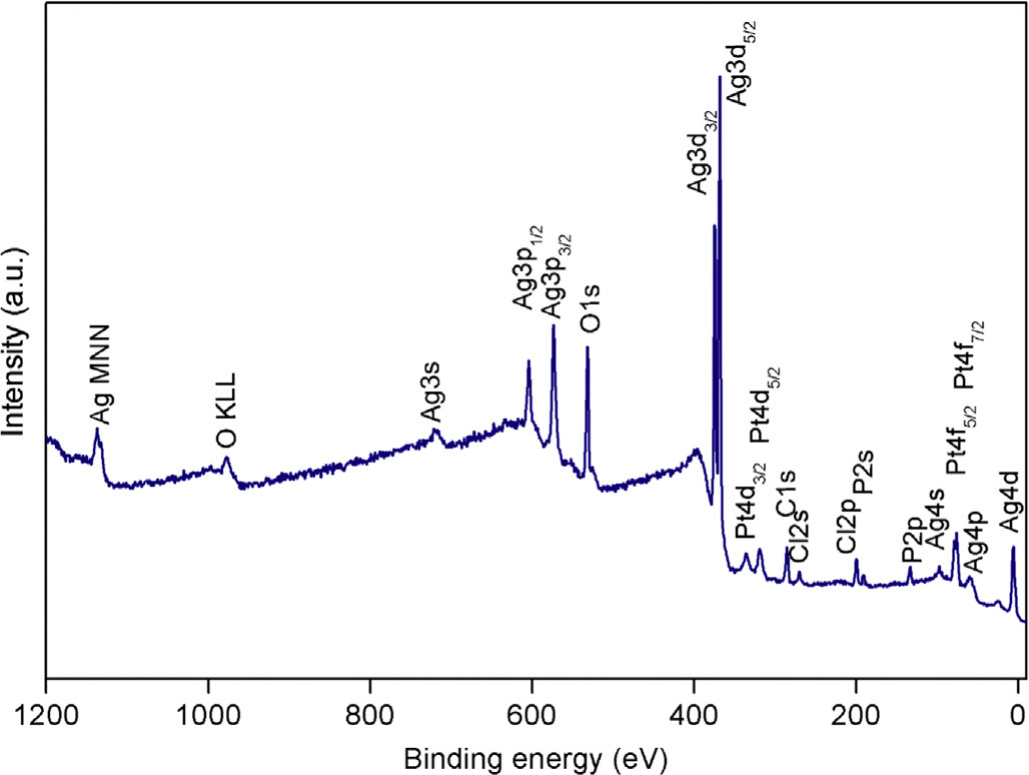
The XPS survey spectrum of defect-Ag_3_PO_4_/PtCl_6_^2−^ (DAP/Pt) synthesized under the co-precipitation method.

**Fig. 2:**
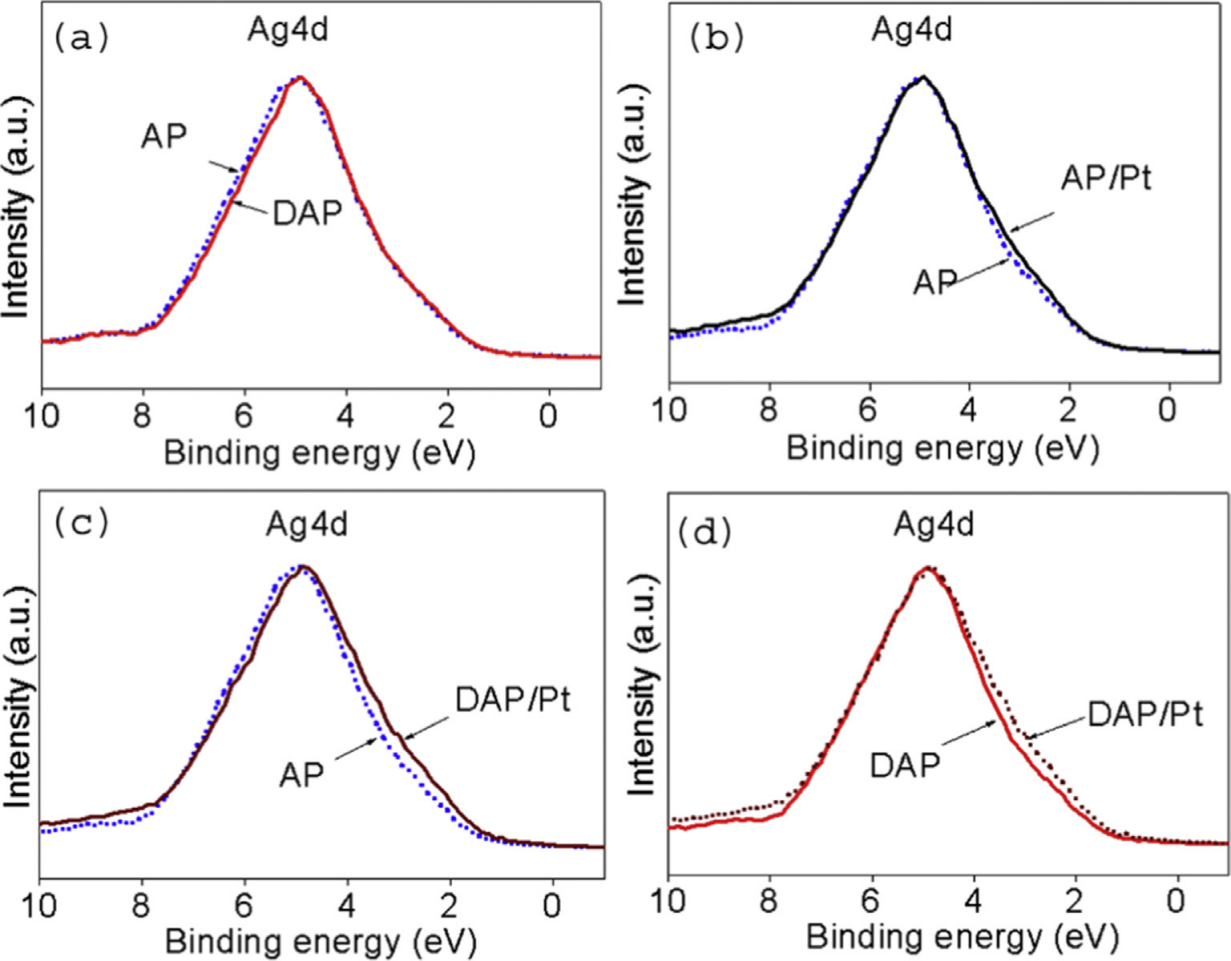
The comparison of the Ag4d spectra of AP to DAP (a), AP/Pt (b), DAP/Pt (c) and comparison of DAP to DAP/Pt (d).

**Fig. 3:**
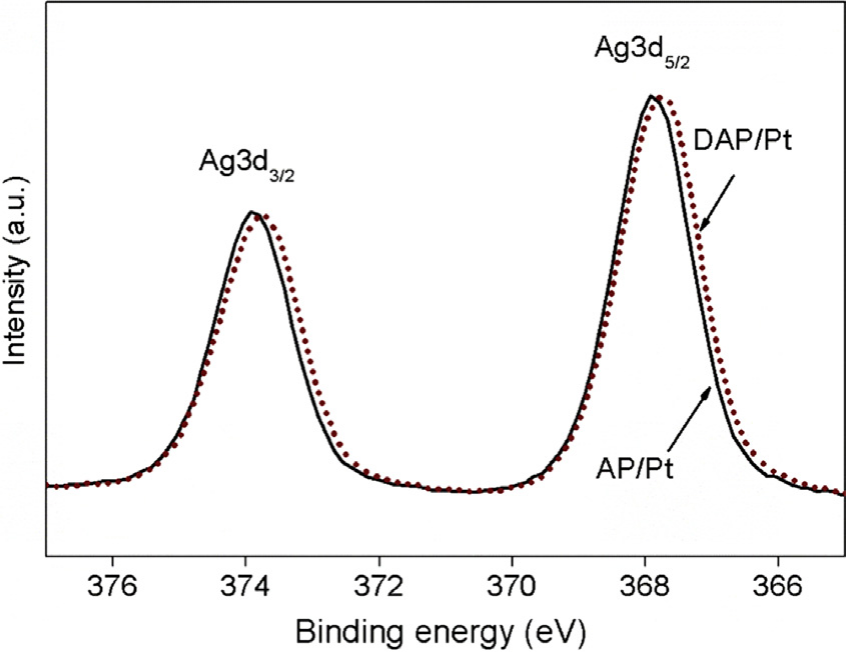
The comparison of the Ag3d spectra of AP/Pt and DAP/Pt.

The XPS survey spectrum of defect-Ag_3_PO_4_/PtCl_6_^2−^(DAP/Pt) was shown in Fig. 1, the dopant of platinum complex anion was observed. The comparison of the Ag4d spectra of AP to DAP, AP/Pt, DAP/Pt, and the comparison of DAP to DAP/Pt are displayed in Fig. 2. A slight peak shrinkage was observed in DAP sample. Doping of PtCl_6_^2−^ to DAP significantly broadened the spectra of Ag4d. It was also found that the binding energies (BEs) of Ag4d decreased significantly after incorporating PtCl_6_^2−^. The BEs of 5.0 eV, 4.9 eV, 4.9 eV, and 4.8 eV were observed for Ag4d in the sample of AP, DAP, AP/Pt, and DAP/Pt, respectively (Table 1). The modification of Ag_3_PO_4_ by defect and dopant changed the energy curve profile of Ag4d. The BEs of 367.8 eV and 373.8 eV were assigned as Ag3d_5/2_ and Ag3d_3/2_, respectively, the silver was in the form of Ag^+^ [3], no metallic silver observed in the samples. The significant shift of Ag3d peak was found in DAP/Pt to AP/Pt (Fig. 3). The defect sites affected the platinum complexes ion dopant in the surface of Ag_3_PO_4_.

**Table 1:** XPS Analysis of AP, DAP, AP/Pt, and DAP/Pt.

Samples	BE Ag3d (eV)	BE Ag4d (eV)	BE P2p (eV)	O-2/O-1
AP	367.8	5.0	132.5	0.25
DAP	367.8	4.9	132.5	0.32
AP/Pt	367.9	4.9	132.7	0.49
DAP/Pt	367.7	4.8	132.7	0.51

**Fig. 4:**
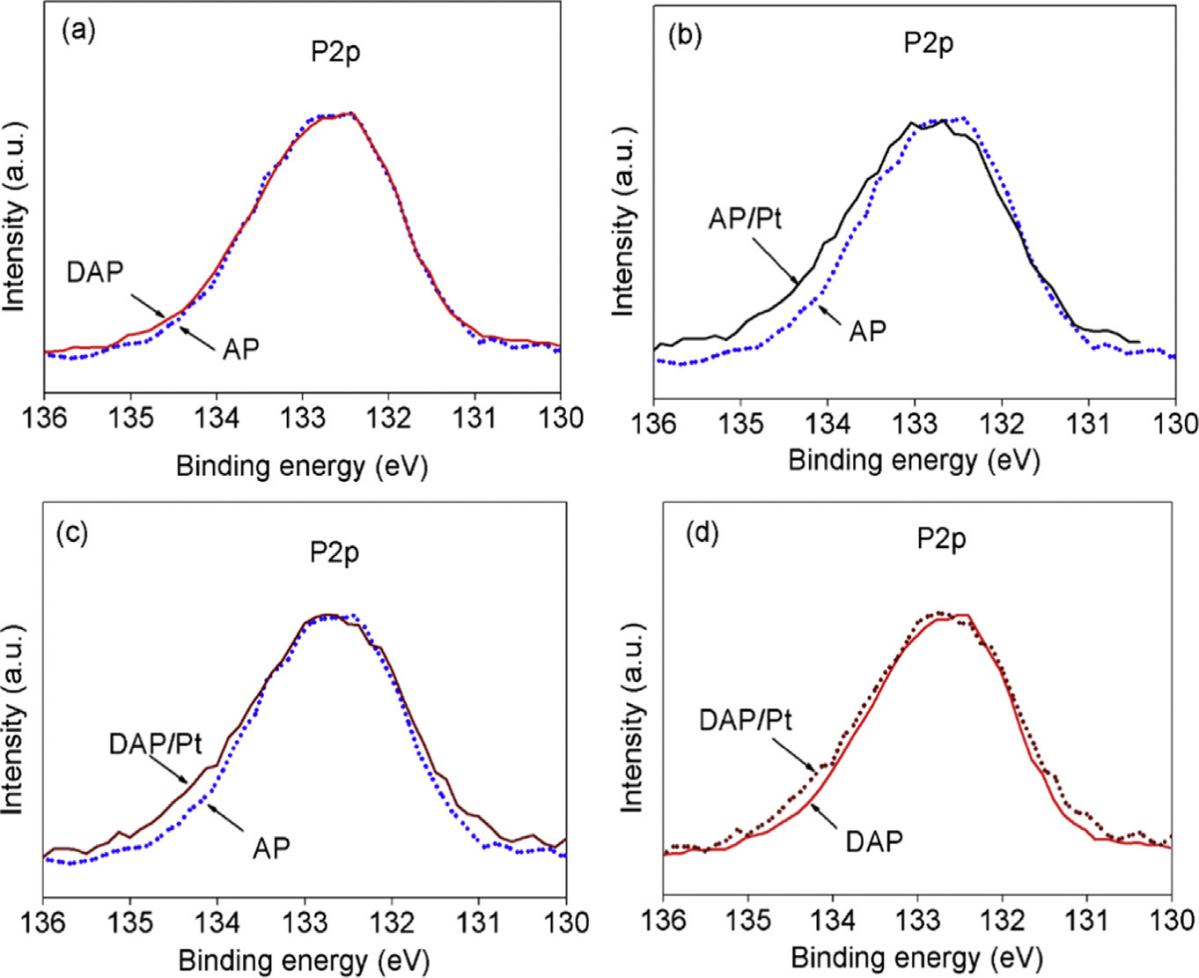
The comparison of the P2p spectra of AP to DAP (a), AP/Pt (b), DAP/Pt (c) and comparison of DAP to DAP/Pt (d).

The BEs P2p of 132.5 eV, 132.5 eV, 132.7 eV, and 132.7 eV were observed for AP, DAP, AP/Pt, DAP/Pt, respectively. These values are originated from the existence of P^5+^ [4,5]. The broaden peak of P2p caused by the platinum complexes ion dopant was observed as shown in Fig. 4.

**Fig. 5:**
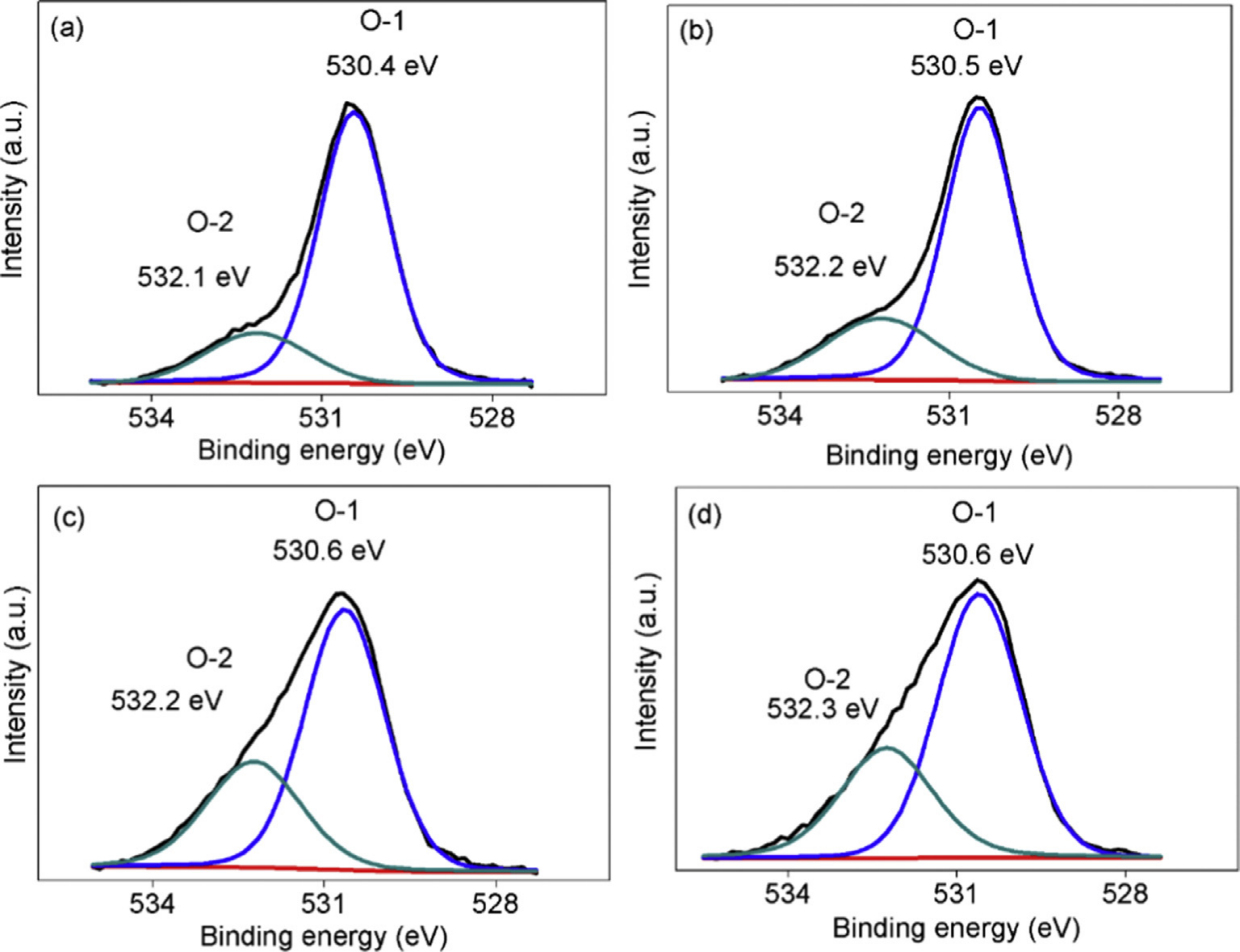
XPS deconvolution of O1s for the sample of (a) Ag_3_PO_4_ (AP), (b) defect-Ag_3_PO_4_ (DAP), (c) Ag_3_PO_4_/PtCl_6_^2−^ (AP/Pt) and (d) defect-Ag_3_PO_4_/PtCl_6_^2−^ (DAP/Pt).

The deconvolution of O1s displayed in Fig. 5. There are two types of oxygen of O-1 and O-2 existed in the surface of Ag_3_PO_4_ with the BE of 530.4 eV and 532.1 eV, respectively. The O-1 can be ascribed to the crystal lattice oxygen whereas the O-2 can be indicated as the surface adsorbed oxygen [6]. After PtCl_6_^2−^ doping, the composition of oxygen was changed. The different ratios of O-2/O-1 were found significantly. The ratios of 0.25, 0.32, 0.49 and 0.51 were found in AP, DAP, AP/Pt, and DAP/Pt, respectively (Table 1). The samples that were incorporated with PtCl_6_^2−^ anion showed a higher ratio of O-2/O-1.

## 2 Experimental design, materials, and methods

The samples of Ag_3_PO_4_, defect-Ag_3_PO_4_, Ag_3_PO_4_/PtCl_6_^2−^ and defect-Ag_3_PO_4_/PtCl_6_^2−^ were named AP, DAP, AP/Pt, and DAP/Pt, respectively. They were prepared by the co-precipitation method [1]. The starting materials of compounds were silver nitrate (AgNO_3_), disodium hydrogen phosphate dodecahydrate (Na_2_HPO_4_.12H_2_O), and hexachloroplatinic acid hexahydrate (H_2_PtCl_6_.6H_2_O). Typically, 0.850 g of AgNO_3_ was dissolved in 200 mL of ethanol-water (1:1), and 1.790 g of Na_2_HPO_4_.12H_2_O was dissolved in 50 mL of water. The Na_2_HPO_4_ aqueous solution was slowly added to AgNO_3_ in ethanol-aqueous solution. The precipitates were filtered and washed with water and dried in an oven at 60 °C for 4 h. This sample was named DAP. To design the platinum complex dopant in DAP, 0.5 g of DAP was suspended in 10 ml of water by sonication. The Pt solution (10 ml) was added to the suspension, then sonicated for 5 minutes followed by mixing under magnetic stirrer for 30 minutes. The Pt solution was made by dissolving of 0.133 g H_2_PtCl_6_.6H_2_O in 100 ml of water solution. The obtained precipitates were filtered and washed with water and dried in an oven at 60 °C for 4 h. This sample was named DAP/Pt. The samples of AP and AP/Pt (defect-free samples) were prepared similarly with this preparation but without ethanol in dissolving of AgNO_3_, only used 200 ml of water.

The four samples prepared were investigated using the XPS instrument (PerkinElmer PHI 5600). The deconvolutions of O1s were analyzed using the software (XPSPEAK Version 4.1).
